# Grape Seed Extract Fortification: Effects on Dough Properties and Quality Attributes of Fresh Wet Noodles from Medium-Gluten Wheat Flour

**DOI:** 10.3390/foods15081400

**Published:** 2026-04-17

**Authors:** Xin Wang, Zengming Gao, Li Yang, Jian Ren, Cuntang Wang

**Affiliations:** 1 College of Food and Bioengineering, Qiqihar University, Qiqihar 161006, China; 2 Engineering Research Center of Plant Food Processing Technology, Ministry of Education, Qiqihar 161006, China; 3 Food Laboratory of Zhongyuan, Luohe 462000, China

**Keywords:** fresh wet noodles, fortification, grape seed extract, antioxidant, medium-gluten wheat flour

## Abstract

The increasing awareness of health among consumers has made the development of functional cereal products a major trend in the food industry. This study investigated the effects of grape seed extract (GSE) on the quality parameters of medium-gluten wheat flour and fresh wet noodles, with the aim of developing functional noodle products. GSE was incorporated at concentrations of 0%, 0.2%, 0.4%, 0.6%, and 1% (*w*/*w*). Its influence on dough properties—including farinographic characteristics, extensibility, and pasting behavior—as well as on noodle quality attributes (antioxidant activity, tensile strength, color, microstructure, total phenolic content, and sensory profile) was evaluated. The results indicated that at 1% GSE addition, the farinographic properties, extensibility, and pasting characteristics of the dough were consistently enhanced. Correspondingly, the noodle microstructure exhibited a more compact and ordered arrangement. Furthermore, increasing the level of GSE fortification led to a significant rise in the total phenolic content and antioxidant capacity of the noodles (*p* < 0.05). This study can provide key technical support for developing novel fresh wet noodle products that possess both excellent quality and antioxidant functionality, thereby contributing to the functional enhancement of staple food products and meeting consumer demand for healthier dietary options.

## 1. Introduction

Amidst a growing global emphasis on health-conscious consumption, the food market is undergoing a profound shift from meeting basic caloric needs toward a dual pursuit of superior quality and nutritional benefits [1]. In response, numerous national initiatives have been implemented to improve public dietary structures, with a common focus on the fortification of staple foods with functional components [2]. Wheat, as one of the world’s three major staple grains, holds a predominant place in global diets, particularly in Asian countries like China and South Korea, where a large proportion of wheat flour is dedicated to noodle production [3]. However, the conventional refining process of wheat often leads to a significant loss of inherent nutrients, resulting in end products that may fall short of modern expectations for nutritional balance [3]. Traditional noodles, typically made from simple ingredients, are notably deficient in several bioactive compounds.

To address this nutritional gap, research has increasingly focused on developing value-added wheat products by supplementing them with natural ingredients to enhance their health profile and appeal [4,5]. Recent advances in food processing technologies have facilitated the development of nutritionally enhanced wheat-based products, including noodles fortified with tea polyphenols [6] or grape seed powder [7], and bread supplemented with grape peel [8] or onion skin extract [9]. For example, Duan et al. [10] demonstrated that the incorporation of lotus rhizome residue into noodle formulations can mitigate cooking loss and enhance noodle expansion rate. Also, it can concurrently attenuate glycemic response. These studies underscore the potential of functional fortification in staple foods.

The quality of fresh wet noodles is fundamentally governed by the protein network formed in the dough, with medium-gluten wheat flour serving as the core raw material. Variations in the glutenin-gliadin equilibrium and the intensity of protein cross-linking exert decisive control over the mechanical and sensory textural properties of the finished noodles, manifesting most notably in their tensile strength, elasticity, and chewiness perception [11]. Nonetheless, noodles made from conventional medium-gluten flour often exhibit limitations, including a less desirable dark yellow color and insufficient antioxidant activity. These shortcomings, coupled with pronounced susceptibility to microbial spoilage—especially in regions with limited cold-chain infrastructure—compromise both shelf life and consumer appeal [12]. Consequently, modulating flour properties through the addition of functional ingredients presents a vital strategy for comprehensively improving noodle quality.

The phenolic compounds in grape seed extract (GSE) are dominated by proanthocyanidins (e.g., proanthocyanidin B1, B2) and flavan-3-ol monomers (catechin, epicatechin), along with phenolic acids and flavonols such as gallic acid and quercetin. Among these, epicatechin and catechin have the highest contents (170 and 88 mg/kg, respectively) [13,14,15]. Its application has been explored in various food matrices, including bread [16] and biscuits [17], demonstrating its efficacy in enhancing phenolic content and extending shelf life [18,19]. Despite these promising bioactive properties, investigations into GSE-fortified noodle systems remain scarce, with available literature yielding equivocal or conflicting findings. In particular, the mechanistic basis underlying GSE-induced modifications to dough physicochemical properties and microstructural architecture has not been fully elucidated, representing a critical gap in the current understanding of phenolic-starch-protein interactions in complex food matrices.

Based on preliminary trials and relevant literature, a GSE concentration range of 0–1.0% (*w*/*w*, flour basis) was selected for this study. Additions above 1.0% resulted in excessive hardness and noticeable bitterness in preliminary screening, thereby reducing sensory acceptability. Therefore, within this concentration range, this study aimed to evaluate the effects of GSE fortification on dough properties and fresh wet noodle quality, and to clarify the potential structure–function relationships of GSE in noodle systems, providing a scientific basis for its value-added application in wheat-based staple foods.

## 2. Materials and Methods

### 2.1. Materials

Materials used in this study mainly included wheat flour from *Triticum aestivum* L. *cv.* Jinmai 108 (without any additives) and grape seed extract (GSE). The medium-gluten wheat flour was purchased from a local supermarket, and the GSE was obtained from Jinfam Microorganism Co., Ltd. (Beijing, China). The GSE was stored in sealed, light-protected containers at −20 °C prior to use.

### 2.2. Tensile Properties of Wheat Dough with GSE

Tensile properties of the dough were determined using an extensograph according to a previous report [20]. Briefly, 300 g of wheat flour containing different levels of GSE was mixed with water in a dough mixer (SM-201/202, Supor Lifestyle Electrical Appliances Co., Ltd. of China (Shaoxing, China)) under the same formulation conditions used for dough preparation. The resulting dough was then molded into a standard cylindrical shape using the tensile tester (JMLD 150, Beijing Dongfu Jiuheng Instrument Technology Co., Ltd., Beijing, China). The dough was placed in a fermentation chamber at 30 °C for 45 min, 90 min, and 135 min before testing to obtain the dough’s extensibility, resistance to extension, and tensile ratio. The test results were the mean of three determinations.

### 2.3. Farinographic Properties of Wheat Flour with GSE

A JFZD 300 farinograph (Beijing Dongfu Jiuheng Instrument Technology Co., Ltd., Beijing, China) was employed to characterize the mixing properties of GSE-enriched doughs, adopting the methodology of Bai-Ngew [21]. The experimental design comprised six GSE inclusion levels (0–1.0%, *w*/*w*, at 0.2% intervals), with 300 g flour samples evaluated per treatment. Instrument settings included: 30 °C operating temperature, 1 min pre-mixing, 61% water addition (flour basis), and 60 r/min blade speed. Automated hydration commenced post pre-mixing, with evaporative losses mitigated via plastic covering throughout the 20 min mixing cycle. The resultant farinograms yielded quality parameters: water absorption, development time, stability, and softening degree. Each formulation was assessed in triplicate under constant instrumental conditions.

### 2.4. Textural Properties of Wheat Flour with GSE

The mechanical properties of dough were characterized using a TA-XTplus texture analyzer (TA-TX PlusC, Stable Micro Systems, Godalming, UK) based on methodology reported by Xie et al. [22] with minor adaptations. Sample preparation involved molding dough into uniform cylinders (30 mm × 35 mm) prior to placement on the instrument stage. All determinations employed a P50 probe under the following operational parameters: pre-contact speed 1.0 mm/s, compression speed 0.8 mm/s, post-compression speed 1.0 mm/s, 70% strain application, 1 s inter-cycle rest period, and 5 g trigger sensitivity. Hardness, chewiness, and elasticity values were extracted from the resulting force-deformation curves. Each experimental condition was analyzed in triplicate.

### 2.5. Pasting Characteristics of Wheat Flour with GSE

Pasting characteristics were assessed with an RVA 4500 instrument (Tianjin Fulu Tong Technology Trading Co., Ltd., Tianjin, China). Sample preparation entailed dispersing flour in distilled water to achieve a 10% (*w*/*w*) concentration, followed by manual agitation (six paddle strokes) to ensure uniform suspension distribution. The canister was then inserted into the instrument for automated analysis following the manufacturer’s specifications. The temperature-time profile consisted of: initial holding (50 °C, 1 min), heating ramp (50–95 °C, 3 min 45 s), high-temperature plateau (95 °C, 2 min 30 s), cooling ramp (95–50 °C, 3 min 45 s), and final holding (50 °C, 2 min) [23]. Instrument-specific software was utilized for data acquisition and calculation of viscographic parameters: peak viscosity, hot paste viscosity, breakdown, final viscosity, setback, pasting temperature, and peak time. All analyses were conducted in triplicate.

### 2.6. Preparation of Noodles

GSE was first dissolved in distilled water at room temperature (25 °C) with stirring for 2 min. The resulting solution was then added to 300 g of wheat flour, and the total water addition was fixed at 180 mL. This level was selected on the basis of preliminary trials to ensure suitable dough cohesiveness, sheeting performance, and noodle formation. The control sample received the same volume of distilled water without GSE. Dough mixing was conducted at low speed for 5 min, followed by high speed for 2 min. The dough was then removed and passed through a pasta machine set to a roller gap of 5 mm. Repeated sheeting was performed until a uniform, compact, and hole-free dough sheet was obtained. The sheet was subsequently cut into fresh noodles using the forming attachment of the pasta machine. To limit oxidative changes, the preparation process was completed within a short period. Part of each noodle sample was freeze-dried under vacuum, ground, passed through a 60-mesh sieve, and stored at −20 °C for subsequent analyses.

### 2.7. Cooking Loss of Noodles

Solid loss during thermal processing was evaluated using an adapted methodology from Wang et al. [24]. Noodle samples (10 g; cut into standardized 10 cm lengths and weighed to ± 0.1 g accuracy) were subjected to boiling in 500 mL deionized water using a glass beaker positioned on an electric hotplate. The cooking duration (5 min) was established as optimal based on the elimination of the central opaque zone. Post-cooking, noodles were separated from the liquid phase, and the extract was cooled to room temperature prior to quantitative transfer into a 500 mL volumetric flask. Following dilution to mark with distilled water, a 50 mL aliquot was evaporated to dryness on a low-temperature hotplate, with subsequent oven drying at 105 °C to constant weight. The percentage cooking loss was derived from the proportion of dried residue relative to the original uncooked noodle dry weight, with triplicate determinations for each sample.

(1) P=5M/G(1−W)×100%

Note: where G is the weight of noodles before cooking, M is the difference between the two constant weights of the beaker, and W is the moisture content of raw noodles.

### 2.8. Water Absorption Rate of Noodles

The water uptake characteristics of noodles were determined following Mojarrad et al. [25]. Cooked specimens were rapidly cooled via immersion in chilled distilled water, surface-adherent water was eliminated by blotting, and samples were subsequently weighed (±0.1 g). Percentage water absorption was calculated as the mass increment during cooking relative to the initial dry mass, expressed mathematically as

(2) $$\mathrm{Water}\;\mathrm{absorption}\;\mathrm{rate}\;(\%)=\frac{W_{f}-W_{i}}{W_{i}}*100$$

where W_i_ and W_f_ denote initial and final weights, respectively.

### 2.9. Breakage Rate of Noodles

Noodle breakage rate was determined following Li et al. [26]. Fifty intact noodle strands were cooked in 500 mL of vigorously boiling water for the optimal cooking time (determined previously). Post-cooking, the number of unbroken strands was counted, and the breakage rate was calculated using the following equation:

(3) $$\mathrm{Breakage}\;\mathrm{rate}\;(\%)=\frac{50-N}{M}*100$$

where N and M denote the number of intact noodles and the total number of noodles, respectively.

### 2.10. Color of Cooked Noodles

The surface color characteristics of noodle specimens were evaluated colorimetrically [27] employing a Linshang Technology instrument (Shenzhen, China). The tristimulus values—L* (lightness), a* (red–green axis), and b* (yellow–blue axis)—were acquired within the CIE Lab* uniform color space under standardized viewing geometry (10° observer angle, CIE standard illuminant). Each reported value constitutes the mean of triplicate measurements.

### 2.11. Tensile Properties of Cooked Noodles

Tensile characteristics of cooked noodles were evaluated utilizing a tensile tester (JMLD 150, Beijing Dongfu Jiuheng Instrument Technology Co., Ltd., Beijing, China) configured in extension mode, following a slightly adapted protocol from Liu et al. [28]. Individual noodle strands with a length of 250 mm were selected for tensile testing after cooking and surface draining. The tensile test was performed using an A/SPR probe, with test speed and post-test speed set at 2.0 mm/s and 2.0 mm/s, respectively. The program-run presetting force was 5.0 g, and the tensile distance was 180 mm. Tensile parameters of the cooked noodles, including tensile strength (TS), elongation (E), and breaking force, were obtained. For each treatment, six repeated tensile measurements were conducted.

### 2.12. Microstructure of Cooked Noodles

Microstructural characterization of noodle surfaces was performed using cold field emission scanning electron microscopy (Gemini 300, Carl Zeiss AG, Oberkochen, Germany). Freeze-dried GSN specimens were mounted on aluminum stubs with the superficial layer oriented upward, secured with conductive adhesive tape, and subsequently subjected to gold sputter coating for 90 s to enhance electron conductivity and reduce charging effects. Imaging was executed at an accelerating voltage of 5 kV and a working magnification of 1000× [24].

### 2.13. Preparation of Polyphenol Extract

Extraction of phenolic constituents was performed by dispersing 1 g portions of raw and cooked noodles (separately weighed) in 10 mL 70% ethanol (*v*/*v*) within conical flasks. The suspensions were subjected to static maceration at room temperature for 24 h. While TPC determination was conducted on all extracts, antioxidant activity assays (DPPH and ABTS) were restricted to cooked noodle extracts to provide complementary radical scavenging profiles.

### 2.14. Total Phenolic Content of Raw and Cooked Noodles

Phenolic analysis was limited to total phenolic content (TPC) determination only. Total phenolic content (TPC) was determined exclusively via the Folin–Ciocalteu colorimetric method [29]. Phenolic concentrations were quantified against a gallic acid standard curve (y = 0.0129x + 0.0103, R^2^ = 0.9988) and expressed as gallic acid equivalents (GAE, mg/100 g dry weight). All analyses were performed in triplicate to ensure reproducibility.

### 2.15. Antioxidant Activity of Cooked Noodles

Antioxidant activity was evaluated using both DPPH and ABTS assays in order to provide complementary information on radical scavenging capacity.

DPPH scavenging activity [30] was assessed by reacting 1 mL of the extract with 4 mL of 0.1 mM DPPH solution. Following 30 min incubation in darkness, absorbance at 517 nm was measured in triplicate.

ABTS^+^· generation followed the protocol of Tuersuntuoheti et al. [30]: 5 mM ABTS (7 mmol·L^−1^) was oxidized with 88 μL potassium persulfate (140 mmol·L^−1^) and aged 12–16 h in darkness. Working solutions were diluted to 0.700 ± 0.02 absorbance units at 734 nm prior to mixing with sample extracts. Percentage inhibition was calculated for both assays using standard formulas.

(4) $$\mathrm{DPPH}/\mathrm{ABTS}\;\mathrm{radical}\;\mathrm{scavenging}\;\mathrm{rate}\;(\%)=\frac{A_{\mathrm{control}}-A_{\mathrm{sample}}}{A_{\mathrm{control}}}*100$$

where A_control_ and A_sample_ denote control absorbance and sample absorbance, respectively.

### 2.16. Sensory Evaluation

Sensory evaluation was carried out using a nine-point hedonic scale according to the method of Adeyemo et al. [31]. The assessed attributes included color, flavor, texture, taste, and overall acceptability, where one indicated “extremely disliked” and nine indicated “extremely liked”. The panel consisted of 30 trained assessors familiar with noodle sensory evaluation. Each sample was coded with a random three-digit number, placed on a white plate, and presented in random order. Panelists were asked to rinse their mouths with warm water before tasting each sample to reduce carryover effects.

### 2.17. Statistical Analyses

All data are presented as mean ± standard deviation (SD). Physicochemical measurements were performed in triplicate (n = 3), except for noodle tensile analysis (n = 6) and sensory evaluation (n = 30). Statistical differences among treatments were analyzed by one-way analysis of variance (ANOVA), followed by Duncan’s multiple range test. Differences were considered significant at *p* < 0.05. Statistical analyses were performed using SPSS 2022 software (IBM Corp., Armonk, NY, USA).

## 3. Results and Discussion

### 3.1. Effect of GSE on Dough Tensile Properties of Medium-Gluten Wheat Flour

**Figure 1:**
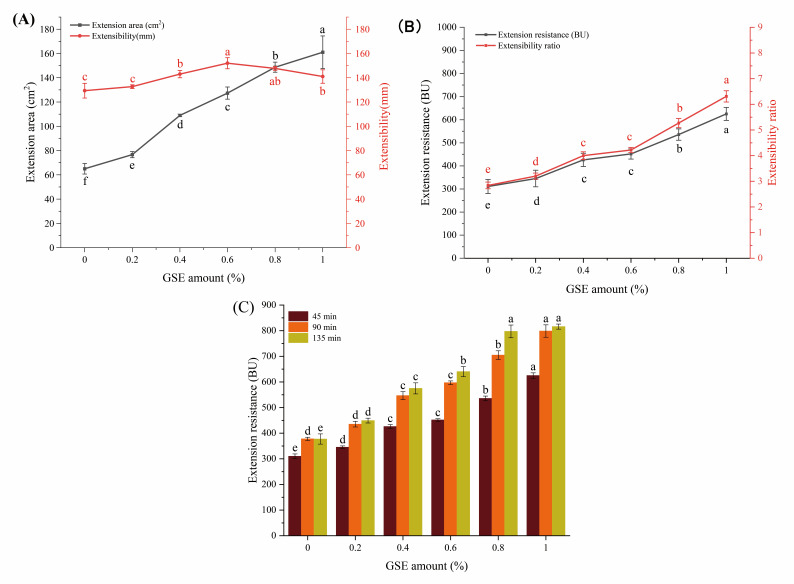
Effect of GSE addition level on dough tensile properties of medium-gluten wheat flour. Note: (**A**) shows extension area and elongation of dough; (**B**) shows extension resistance and extensibility ratio of dough; (**C**) shows extension resistance of dough at different resting times (45, 90, 135 min). Different lowercase letters indicate significant differences (*p* < 0.05).

Dough tensile properties are core indicators for evaluating processing adaptability and textural properties of end products, primarily reflecting the mechanical response capacity of the gluten protein network and the intensity of intermolecular interactions [32]. Figure 1A shows that with the increase in the addition level of grape seed extract (GSE), the extension area expands from 65.00 cm^2^ to 161.00 cm^2^, while the extensibility first increases from 129.00 mm to 152.00 mm and then decreases to 141.00 mm. Figure 1B demonstrates that the extension resistance significantly increases from 311.00 BU to 625.00 BU, and the extensibility ratio rises from 2.80 to 6.30. Extensibility refers to the maximum deformation length of the dough from the start of stretching to fracture under external force. The change in extensibility suggests that the reducing properties of polyphenolic compounds in GSE may react with oxidative intermediates in the dough, consuming oxidants that promote disulfide bond formation, thereby slowing down disulfide bond formation and limiting dough extensibility. The inclusion of GSE positively enhanced the dough’s tensile and elastic properties. Such improvement is advantageous for further processing [33].

The variation in extension resistance at different resting times (45, 90, 135 min) (Figure 1C) provides clearer insights. Extension resistance increased with resting time, indicating greater resistance to extension after resting. In conclusion, the quality of dough supplemented with GSE was improved, with enhanced tensile and elastic properties of fresh dough, which also benefits baking and processing performance. Notably, GSE-added dough exhibited similar changes in extensigraph resistance to anthocyanin-added dough [34].

### 3.2. Effect of GSE on Textural and Farinographic Properties of Medium-Gluten Wheat Dough

**Figure 2:**
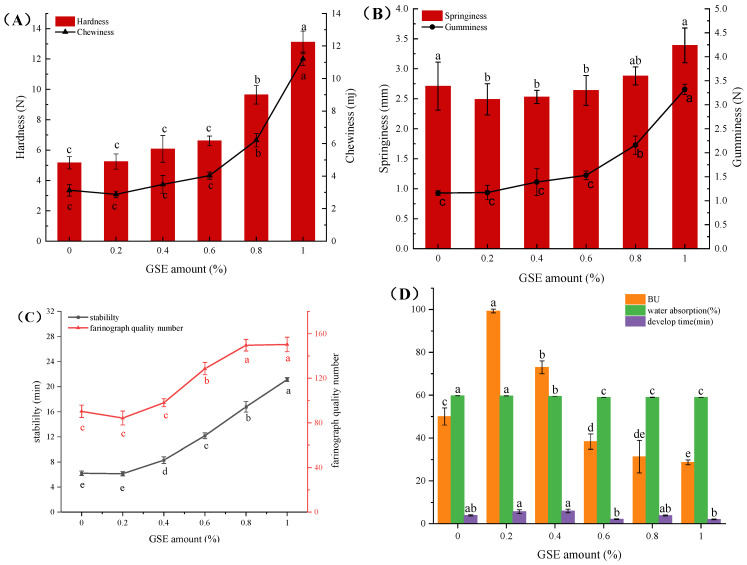
Effects of GSE addition level on the textural (**A**,**B**) and farinographic (**C**,**D**) properties of medium-gluten wheat dough. (**A**) Hardness and chewiness; (**B**) springiness and gumminess; (**C**) stability time and farinograph quality number; (**D**) degree of softening, water absorption, and development time. Values with different lowercase letters denote significant differences (*p* < 0.05).

Hardness, gumminess, springiness, and chewiness are critical parameters of noodle textural properties, which are closely associated with the palatability of noodles [35]. Texture profile analysis (TPA) simulates the secondary chewing process, and TPA indices show significant correlation with sensory evaluation results. Textural parameters of dough with different GSE concentrations were measured and summarized in Figure 2A,B. Results showed that the hardness, chewiness, and gumminess of dough significantly increased with GSE addition, whereas springiness exhibited a trend of first decreasing and then increasing. This phenomenon can be attributed to the interactions between the polyphenolic compounds in grape seed extract and gluten proteins. The abundant hydroxyl groups in GSE polyphenols (proanthocyanidins) can form hydrogen bonds with polar amino acid residues (e.g., glutamine, serine) in gluten proteins, while their aromatic rings engage in hydrophobic interactions with exposed hydrophobic amino acid residues. At higher GSE concentrations, oxidized polyphenol intermediates (quinones) may form covalent cross-links with sulfhydryl groups (-SH) of cysteine or amino groups (-NH_2_) of lysine residues, thereby further stabilizing the gluten network structure [36,37,38].

Although farinographic analysis is traditionally used in bread dough research, its parameters are also informative for noodle systems because they reflect dough hydration, development, mixing tolerance, and structural stability during processing. Farinographic experiments (Figure 2C,D) revealed that the dough development time reached its maximum at GSE addition levels of 0.2% and 0.4%, while it decreased to its minimum at addition levels of 0.6% and 1.0%. Meanwhile, stability time was significantly prolonged, while the degree of softening varied depending on the GSE level. At low GSE levels, polyphenols might interfere with the formation of gluten networks, leading to prolonged development time, increased degree of softening, and decreased springiness. With higher GSE addition, the stabilizing effect of polyphenols on gluten proteins gradually intensified, prolonging stability time, reducing the degree of softening, enhancing hardness and chewiness, and recovering springiness. Phenolic compounds may influence gluten network formation through both competitive binding and cross-linking-related mechanisms. At lower levels, they may compete with proteins for water and interfere with early protein aggregation, whereas at higher levels they may enhance structural stability through hydrogen bonding, hydrophobic interactions, and possible quinone-mediated covalent reactions [39]. Similar concentration-dependent effects of plant polyphenols on dough development and gluten-related properties have also been reported in wheat dough and noodle systems enriched with onion skin extracts, tea polyphenols, and anthocyanins, supporting the view that polyphenol–protein interactions are key determinants of dough behavior [11,33,34].

### 3.3. Effect of GSE on Pasting Properties of Medium-Gluten Wheat Flour

The viscographic characteristics of flour–GSE composites (Table 1) are indicative of starch hydration dynamics and macromolecular interactions during the heating cycle [40]. Notably, peak viscosity exhibited a concentration-dependent decrease following GSE supplementation, declining from 555.33 cP (control) to 546.33 cP (1.0% GSE). This attenuation presumably arises from competitive interactions between GSE polyphenols and starch granules, which disrupt the protein–starch matrix and consequently diminish viscosity development during the pasting process. Such GSE-induced modifications to pasting profiles align with previous observations in phenolic-enriched cereal systems [41,42]. Pasting temperature did not differ significantly among treatments, indicating that the onset of gelatinization was not markedly affected by GSE under the present conditions. Likewise, although breakdown values varied among samples, no clear statistically consistent trend was observed. By contrast, setback values increased at higher GSE levels, which may suggest enhanced molecular reassociation during cooling. This behavior could be related to starch–polyphenol interactions that influence the retrogradation tendency of the flour system [43,44,45].

### 3.4. The Cooking Properties of GSE Noodles

Cooking loss reflects the degree of noodle damage and the ability of the noodle structure to maintain its integrity during cooking. As shown in Table 2, GSE addition generally reduced the breakage rate and cooking loss of fresh wet noodles, while increasing water absorption, although the magnitude of these changes depended on the supplementation level. The reduced breakage rate and cooking loss may be attributed to the presence of multiple phenolic hydroxyl groups in GSE polyphenols, which may strengthen the gluten network through interactions with gluten proteins. A stronger and more compact network can better withstand external forces during processing and cooking, thereby reducing noodle breakage and limiting the loss of soluble solids [46]. In addition, GSE contains abundant hydrophilic groups, such as hydroxyl groups, which may enhance water binding through hydrogen-bond formation with water molecules [47]. As a result, the water absorption of noodles increased with GSE addition and tended to level off at higher supplementation levels. This finding suggests that GSE may have influenced water binding and moisture distribution within the starch–protein matrix [48]. The reduction in cooking loss was also likely associated with improved gluten matrix stability and changes in starch-related behavior. A more compact gluten network could better retain starch granules and soluble components during cooking, while altered starch gelatinization and starch–water interactions may also have contributed to reduced solid loss. These findings are consistent with previous reports on polyphenol- or plant powder-enriched noodles, in which improved network compactness and modified starch–water interactions were associated with reduced cooking loss and altered water absorption behavior [49,50,51].

### 3.5. The Effect of GSE on the Color of Noodles

**Figure 3:**
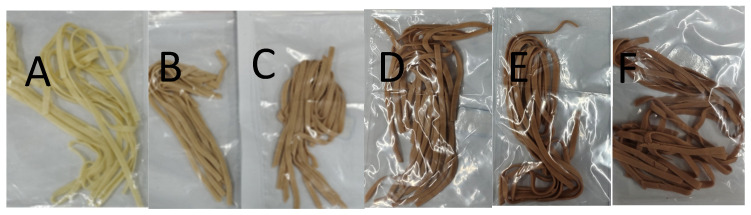
Representative appearance of noodles fortified with different levels of GSE: (**A**) control (0% GSE), (**B**) 0.2% GSE, (**C**) 0.4% GSE, (**D**) 0.6% GSE, (**E**) 0.8% GSE, and (**F**) 1.0% GSE (*w*/*w*, flour basis).

**Figure 4:**
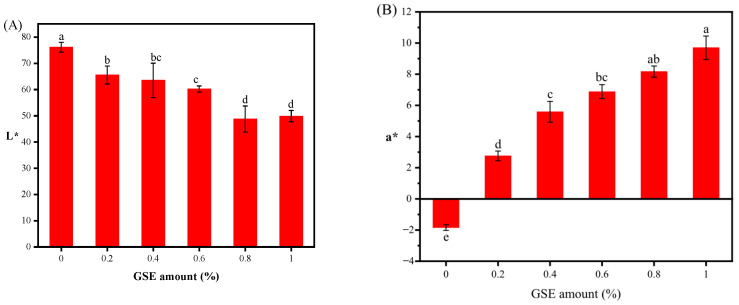

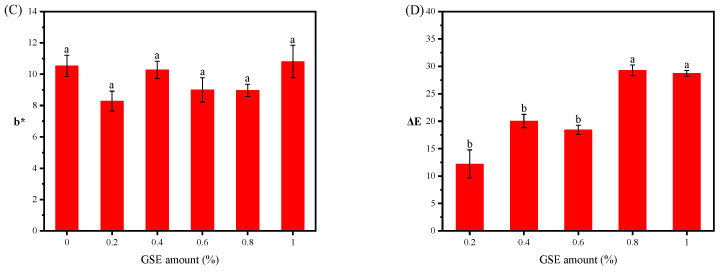
Effects of GSE addition amount on noodle color. Notes: (**A**) represents noodle lightness; (**B**) represents noodle yellow–green ratio; (**C**) represents noodle yellow–blue ratio; (**D**) represents noodle color difference. Different English letters indicate significant differences in values (*p* < 0.05).

Color is considered a major factor determining the marketability of fresh noodles, and it also indicates the raw material quality and shelf life of the product [52]. Noodle color was evaluated using the CIELAB color system, and the L*, a*, and b* values were recorded for each sample. Representative photographs of noodles fortified with different levels of GSE are shown in Figure 3, illustrating the gradual visual transition from light yellow to brownish-red as the GSE level increased. As shown in Figure 4, with the increase in GSE content, the noodle lightness (L*) decreased, the a* value increased significantly, the b* value showed no obvious change, and the color difference (ΔE) value increased, causing the noodles to appear brownish-red. This color change in noodles is related to the polyphenolic compounds in GSE, which may undergo complexation reactions with proteins, starches, etc., in the noodles to produce other colored substances, thereby affecting the noodle color. The brownish-red appearance of GSE-fortified noodles is likely related, at least in part, to the intrinsic color of GSE, although the color parameters of the GSE powder itself were not measured in this study [48].

### 3.6. The Tensile Properties of GSE Noodles

**Figure 5:**
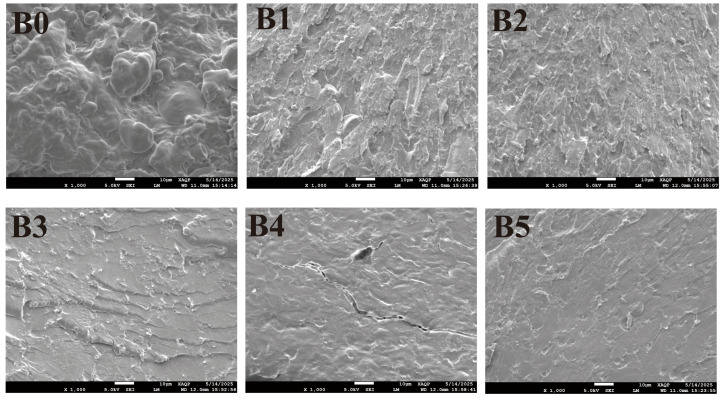
Scanning electron microscope images of noodles with different grape seed extract additions. Notes: (**B0**–**B5**) represent noodles prepared by replacing medium-gluten wheat flour with 0%, 0.2%, 0.4%, 0.6%, 0.8%, and 1% grape seed extract, respectively.

The tensile properties of noodles are key indicators for evaluating their structural integrity and texture quality, which are inherently linked to the molecular architecture of the gluten network and starch-protein interactions [53]. As shown in Table 3, the addition of GSE to flour resulted in an upward trend in extensibility but a downward trend in breaking force of fresh wet noodles with increasing GSE content. Specifically, the extensibility increased from 18.29 mm in the control group to a final 28.28 mm, while the breaking force decreased from 48.89 g to 39.63 g, accompanied by an upward trend in tensile strength (TS). These macroscopic changes corresponded to microstructural transformations observed by SEM (Figure 5). The gluten network evolved from a loose, disorganized structure to a compact, continuous matrix with uniformly embedded starch granules. This denser architecture enables the noodle to undergo greater tensile deformation (higher extensibility) and distribute mechanical stress more effectively (higher tensile strength) before fracture. This is because when the content of polyphenolic compounds in grape seed extract is low, moderate cross-linking occurs, improving and stabilizing the gluten network structure. At moderate GSE levels, polyphenol–protein interactions may contribute to a more compact and continuous gluten–starch matrix, which improves stress distribution during tensile deformation and enhances noodle extensibility and tensile strength [54].

### 3.7. The Microstructure of GSE Noodles

To determine the effect of GSE on the development of the gluten network in noodle systems, the microstructure of cooked noodles with different GSE additions was observed using scanning electron microscopy (SEM), and their microstructures were characterized. As shown in Figure 5B0, the control sample exhibited a relatively loose gluten network with starch granules distributed disorderly. In contrast, the GSE-fortified samples (Figure 5B1–B5) showed progressively smoother and more compact structures with increasing GSE addition. SEM analysis revealed that GSE addition progressively transformed the gluten–starch matrix from a loose and disorganized structure into a more compact and continuous network. This may be because GSE can better fill the gluten protein network structure, and the polyphenolic substances contained in it interact with gluten proteins to promote the formation of the gluten network [54]. This microstructural observation is in good agreement with the tensile results, indicating that GSE-induced reinforcement of the gluten–starch matrix likely contributed to improved resistance to tensile deformation.

### 3.8. The Total Phenolic Content of GSE Noodles

**Figure 6:**
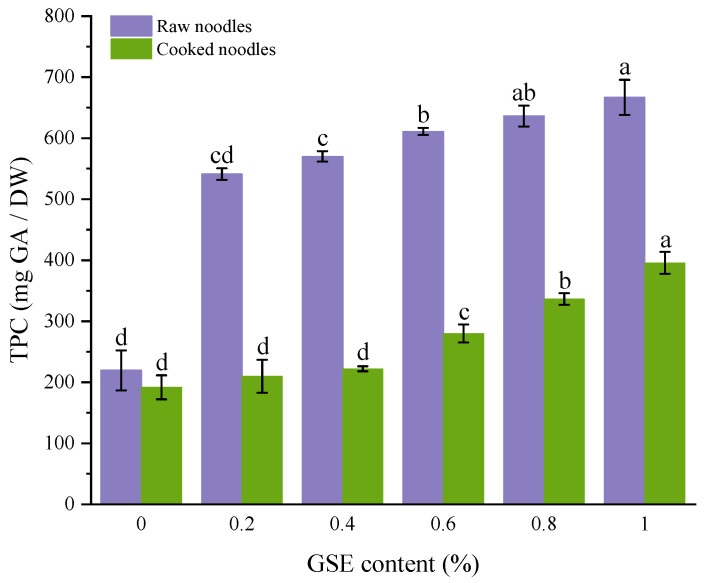
Effects of GSE addition amount on total phenolic content in noodles. Different lowercase letters indicate significant differences in values (*p* < 0.05).

Raw and cooked noodles were both analyzed for TPC in order to assess phenolic retention after the cooking process. As shown in Figure 6, the total phenolic content (TPC) in raw noodles increased significantly after GSE addition, rising from 219.36 mg GAE/100 g DW in the control group to 666.72 mg GAE/100 g DW in the 1% addition group, which was three times that of the control. This is because during noodle processing, the formation of the protein network may fix polyphenolic molecules through hydrogen bonding and hydrophobic interactions, while the steric hindrance effect between starch molecules may help reduce phenolic loss during noodle processing and cooking [55]. However, the TPC of cooked noodles was significantly lower than that of raw noodles, which may be due to the oxidation of phenolic compounds under high temperatures during cooking, leading to polyphenol loss [11]. Similar observations have been reported in other food systems, where pretreatment, thermal processing, and storage conditions significantly affected oxidation-related parameters and the retention of phenolic compounds and antioxidant activity [56,57].

### 3.9. The Antioxidant Activity of GSE Noodles

**Figure 7:**
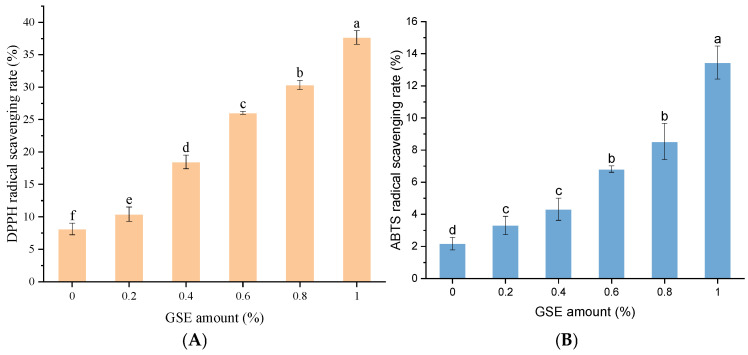
Effects of GSE addition amount on antioxidant activity of noodles. Notes: (**A**) represents the DPPH radical scavenging rate of noodles; (**B**) represents the ABTS radical scavenging rate of noodles. Different English letters indicate significant differences in values (*p* < 0.05).

Figure 7 illustrates the antioxidant characteristics of experimental noodles, with congruent results obtained from both DPPH and ABTS^+^· scavenging methodologies. Phenolic antioxidants function through a structural basis of hydroxyl-substituted aromatic frameworks that stabilize phenoxy radicals via resonance delocalization [58]. These bioactive compounds manifest protective effects through pluralistic mechanisms: primarily, redox-based inactivation of free radicals and their precursors through electron or hydrogen atom donation, or alternatively, prophylactic inhibition of radical initiation [59]. Supplementary protection derives from metal ion sequestration, which curtails hydroxyl radical formation via Fenton reaction suppression [60]. Previous studies on plant-derived bioactive systems have also shown that processing and pretreatment conditions can markedly influence phenolic stability, antioxidant activity, and oxidation-related quality changes, further supporting the importance of considering these factors in GSE-fortified noodle systems [56,57,61].

Figure 7A,B illustrates that GSE fortification engendered marked enhancements in both DPPH and ABTS radical scavenging capacities. Quantitatively, elevating GSE concentrations from 0.2% to 1.0% elicited progressive increases in scavenging efficiencies: DPPH activity surged from 10.41% to 37.67%, while ABTS values climbed from 3.31% to 13.45%. These results confirm that phenolic constituents transferred from GSE to the noodle matrix retain significant antioxidant functionality. The strong positive correlation between total phenolic content and radical scavenging efficacy (evidenced by parallel dose-dependent trajectories across both assays) suggests that phenolic compounds serve as the primary determinant of antioxidant capacity in these fortified products.

### 3.10. Sensory Evaluation of GSE Noodles

**Figure 8:**
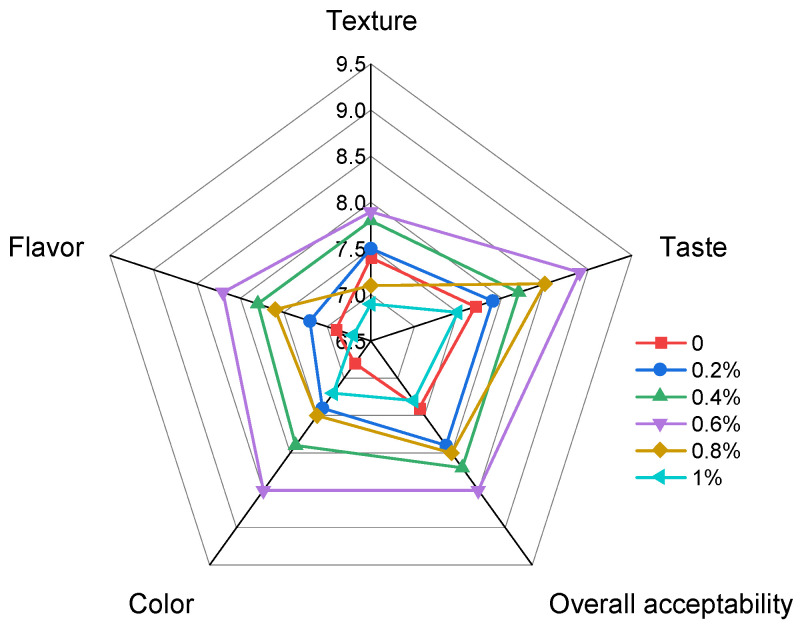
Radar chart illustrating sensory profiles of fresh wet noodles fortified with varying concentrations of grape seed extract (GSE). Values represent mean scores (n = 30) evaluated using a 9-point hedonic scale (1 = dislike extremely, 5 = neither like nor dislike, 9 = like extremely).

Sensory evaluation indices for noodles include flavor, color, taste, texture, and overall acceptability. Consistent with previous studies on noodle quality evaluation, sensory attributes remain essential for assessing consumer acceptance [40,62]. Adverse effects of GSE addition on these attributes may lead to customer rejection, highlighting the critical role of sensory analysis in new product development [50]. The sensory evaluation results are presented in Figure 8 and Table 4.

Compared with the control, noodles containing 0.4–0.8% GSE generally received higher sensory scores, especially in color, flavor, and overall acceptability. Among all treatments, the 0.6% GSE sample obtained the highest scores for color (8.5), flavor (8.2), taste (8.9), and overall acceptability (8.5), and these values were significantly higher than those of the control (*p* < 0.05). The scoring framework designated 5 as the indifference point (“neither like nor dislike”), with higher values indicating favorable reception and lower values unfavorable reception. Figure 8 demonstrates universal acceptance across all samples, as evidenced by scores exceeding the neutral midpoint. Importantly, the brownish-red color induced by GSE addition did not negatively affect consumer acceptance at moderate supplementation levels. Samples containing 0.4–0.8% GSE received comparable or slightly higher color scores than the control, indicating that the color change was visually acceptable to the panelists.

## 4. Conclusions

Our findings demonstrate that grape seed extract (GSE) serves as an effective functional modifier for fresh wet noodle applications. GSE fortification simultaneously enhanced dough processability (stability), noodle texture (extensibility, tensile strength), and hydration properties, effects attributed to the induced formation of a dense, continuous gluten–starch matrix. Concurrently, GSE delivered significant nutritional value through elevated phenolic content and antioxidant activity. Critically, these benefits were achieved without compromising sensory quality at moderate inclusion levels. This study establishes GSE as a promising clean-label functional ingredient for developing premium fresh noodles with enhanced nutritional and technological attributes.

In addition to antioxidant activity, the quality improvement induced by GSE may be related to several physicochemical mechanisms, including polyphenol–protein interactions, starch–polyphenol complex formation, and altered water binding behavior within the noodle matrix. However, several limitations should be noted. The phenolic composition of the tested GSE was not characterized at the individual-compound level, and phenolic retention during different processing stages was not systematically evaluated. In addition, dynamic rheology, direct water-distribution analysis, and multivariate statistical analysis were not performed. Future studies should therefore focus on compound-level phenolic characterization, molecular interaction mechanisms, storage stability, and the optimization of GSE fortification under industrial processing conditions.

## Figures and Tables

**Table 1:** Effect of GSE addition levels on gelatinization properties of medium-gluten wheat flour.

	Pasting Temperature (°C)	Peak Viscosity (cp)	Break Down (cp)	Setback (cp)
Control	61.03 ± 1.89 ^a^	555.33 ± 8.74 ^ab^	117.00 ± 5.29 ^ab^	428.33 ± 2.52 ^d^
0.2%	60.23 ± 1.62 ^a^	572.00 ± 7.55 ^a^	112.33 ± 7.51 ^ab^	449.33 ± 13.20 ^bc^
0.4%	60.20 ± 1.92 ^a^	574.33 ± 8.02 ^a^	109.00 ± 5.00 ^b^	447.00 ± 4.58 ^bc^
0.6%	60.50 ± 0.89 ^a^	564.67 ± 3.06 ^ab^	114.67 ± 1.53 ^ab^	436.33 ± 7.51 ^cd^
0.8%	58.70 ± 1.41 ^a^	576.33 ± 22.05 ^a^	123.00 ± 12.00 ^a^	452.00 ± 7.21 ^b^
1%	60.63 ± 0.93 ^a^	546.33 ± 11.59 ^b^	115.67 ± 5.51 ^ab^	465.67 ± 5.51 ^a^

Note: Data are presented as mean ± standard deviation (SD). Different letters in the same column indicate significant differences among treatments (*p* < 0.05).

**Table 2:** Impact of GSE on cooking characteristics of fresh wet noodles.

	Broken Rate (%)	Cooking Loss (%)	Water Absorption (%)
Control	19.71 ± 1.02 ^a^	17.21 ± 0.98 ^a^	161.90 ± 12.13 ^d^
0.2%	18.64 ± 0.99 ^b^	16.67 ± 0.37 ^a^	196.04 ± 10.23 ^c^
0.4%	17.76 ± 1.21 ^bc^	13.27 ± 0.19 ^b^	248.03 ± 13.19 ^b^
0.6%	16.00 ± 1.89 ^c^	12.26 ± 1.09 ^bc^	265.30 ± 16.10 ^b^
0.8%	13.53 ± 0.78 ^d^	8.38 ± 0.18 ^d^	346.43 ± 10.89 ^a^
1%	10.29 ± 0.12 ^e^	7.61 ± 2.91 ^d^	346.08 ± 13.27 ^a^

Notes: Values represent mean ± standard deviation. Different letters within a column denote significant differences (*p* < 0.05).

**Table 3:** Impact of GSE on tensile properties of fresh wet noodles.

	Extensibility (mm)	TS (Mpa)	Breaking Force (g)
Control	18.29 ± 2.52 ^d^	0.24 ± 0.03 ^c^	48.89 ± 4.12 ^a^
0.2%	23.47 ± 2.33 ^c^	0.28 ± 0.01 ^b^	45.13 ± 1.75 ^b^
0.4%	23.97 ± 4.84 ^c^	0.28 ± 0.01 ^b^	42.75 ± 1.19 ^c^
0.6%	24.04 ± 3.04 ^bc^	0.29 ± 0.02 ^b^	41.96 ± 2.38 ^d^
0.8%	25.56 ± 2.50 ^b^	0.29 ± 0.01 ^b^	40.12 ± 1.15 ^e^
1%	28.28 ± 2.90 ^a^	0.32 ± 0.02 ^a^	39.63 ± 2.33 ^f^

Notes: Values represent mean ± standard deviation. Different letters within a column indicate significant differences (*p* < 0.05).

**Table 4:** Sensory evaluation of fresh wet noodles with varying GSE concentrations.

	Color	Flavor	Texture	Taste	Overall Acceptability
Control	6.8 ± 0.98 ^f^	6.9 ± 0.32 ^e^	7.4 ± 0.39 ^b^	7.7 ± 0.54 ^e^	7.4 ± 0.45 ^e^
0.2%	7.4 ± 0.56 ^d^	7.2 ± 0.98 ^d^	7.5 ± 1.04 ^b^	7.9 ± 1.91 ^d^	7.9 ± 1.11 ^c d^
0.4%	7.9 ± 1.09 ^b^	7.8 ± 0.72 ^b^	7.8 ± 0.89 ^a^	8.2 ± 0.61 ^c^	8.2 ± 0.50 ^b^
0.6%	8.5 ± 1.04 ^a^	8.2 ± 1.01 ^a^	7.9 ± 1.02 ^a^	8.9 ± 0.11 ^a^	8.5 ± 0.12 ^a^
0.8%	7.5 ± 0.50 ^c^	7.6 ± 0.39 ^c^	7.1 ± 0.77 ^c^	8.5 ± 0.79 ^b^	8.0 ± 0.43 ^c^
1%	7.2 ± 1.90 ^e^	6.7 ± 1.12 ^f^	6.9 ± 0.24 ^d^	7.5 ± 1.01 ^f^	7.3 ± 1.23 ^e^

Notes: Data represent mean ± standard deviation (*n* = 30). Different superscript letters within a column indicate significant differences (*p* < 0.05).

## Data Availability

The original contributions presented in this study are included in the article. Further inquiries can be directed to the corresponding authors.

## References

[1] Cao W., Chen J., Li L., Ren G., Duan X., Zhou Q., Zhang M., Gao D., Zhang S., Liu X. (2022). Cookies Fortified with *Lonicera japonica* Thunb. Extracts: Impact on Phenolic Acid Content, Antioxidant Activity and Physical Properties. Molecules.

[2] Boinwad D.L., Shinde R.S. (2024). Health and Wellness Food Products: A Retailers’ perspective. Int. J. Sci. Res. Sci. Technol..

[3] Han C., Ma M., Zhang H., Li M., Sun Q. (2019). Progressive study of the effect of superfine green tea, soluble tea, and tea polyphenols on the physico-chemical and structural properties of wheat gluten in noodle system. Food Chem..

[4] Zhang M., Peng H., Li B., Tian J. (2023). Impact of pomegranate fruit powder on dough, textural and functional properties of fresh noodle. J. Sci. Food Agric..

[5] Xie L., Zhou W., Zhao L., Peng J., Zhou X., Qian X., Lu L. (2023). Impact of okara on quality and in vitro starch digestibility of noodles: The view based on physicochemical and structural properties. Int. J. Biol. Macromol..

[6] Li H., Ma J., Zhao B., Pan L., Meng J., Xu B. (2020). Effect of tea polyphenols on the quality characteristics of fresh wheat noodles in the storage. Int. J. Food Sci. Technol..

[7] Cercel F., Burluc R.M., Alexe P. (2016). Nutritional effects of added fish proteins in wheat flour bread. Agric. Agric. Sci. Procedia.

[8] Bhatt S., Gupta M. (2023). Formulation of instant noodles incorporated with insoluble dietary fiber from fruit peel: In vitro starch digestibility, biophysical, structural and textural characteristics. Appl. Food Res..

[9] Wang C., Wang Y., Wang N., Ren J. (2025). Influence of Onion Peel Extract on the Dough Characteristics of High-Gluten Wheat Flour and the Quality of Bread. Foods.

[10] Duan R., Huang Z., Chen X., Liu Y., Li J., Yan S. (2025). Effect of lotus rhizome residue on the quality and nutritional properties of wheat-based noodles. J. Food Sci..

[11] Wang Y., Jiao R., Wang N., Wang C. (2025). Effects of yellow onion skins extract on the structure and physicochemical properties of fresh noodles and gluten protein. LWT.

[12] Li M., Zhu K.-X., Wang B.-W., Guo X.-N., Peng W., Zhou H.-M. (2012). Evaluation the quality characteristics of wheat flour and shelf-life of fresh noodles as affected by ozone treatment. Food Chem..

[13] Meral R., Doğan İ.S. (2013). Grape seed as a functional food ingredient in bread-making. Int. J. Food Sci. Nutr..

[14] Kuchtová V., Kohajdová Z., Karovicova J., Lauková M. (2018). Physical, textural and sensory properties of cookies incorporated with grape skin and seed preparations. Pol. J. Food Nutr. Sci..

[15] Manca M.L., Casula E., Marongiu F., Bacchetta G., Sarais G., Zaru M., Escribano-Ferrer E., Peris J.E., Usach I., Fais S. (2020). From waste to health: Sustainable exploitation of grape pomace seed extract to manufacture antioxidant, regenerative and prebiotic nanovesicles within circular economy. Sci. Rep..

[16] García-Lomillo J., González-SanJosé M.L. (2017). Applications of wine pomace in the food industry: Approaches and functions. Compr. Rev. Food Sci. Food Saf..

[17] Pasqualone A., Bianco A.M., Paradiso V.M., Summo C., Gambacorta G., Caponio F. (2014). Physico-chemical, sensory and volatile profiles of biscuits enriched with grape marc extract. Food Res. Int..

[18] Iuga M., Mironeasa S. (2020). Potential of grape byproducts as functional ingredients in baked goods and pasta. Compr. Rev. Food Sci. Food Saf..

[19] Karnopp A.R., Figueroa A.M., Los P.R., Teles J.C., Simões D.R.S., Barana A.C., Kubiaki F.T., de Oliveira J.G.B., Granato D. (2015). Effects of whole-wheat flour and bordeaux grape pomace (*Vitis labrusca* L.) on the sensory, physicochemical and functional properties of cookies. Ciênc. Tecnol. Aliment..

[20] Deng X., Chang X., Chen L., Ding W., Wang Y., Li J., Hao Z. (2023). Ultrasonic-assisted resting of Tartary buckwheat dough: Study on its effect and mechanism. Ultrason. Sonochem..

[21] Bai-Ngew S., Therdthai N., Zhou W. (2021). Microwave vacuum-dried durian flour and its application in biscuits. Heliyon.

[22] Xie X., Cai K., Yuan Z., Shang L., Deng L. (2022). Effect of Mealworm Powder Substitution on the Properties of High-Gluten Wheat Dough and Bread Based on Different Baking Methods. Foods.

[23] Chen L., Tong Q., Ren F., Zhu G. (2014). Pasting and rheological properties of rice starch as affected by pullulan. Int. J. Biol. Macromol..

[24] Wang J., Zhang T., Guan E., Zhang Y., Wang X. (2024). Physicochemical properties of wheat granular flour and quality characteristics of the corresponding fresh noodles as affected by particle size. LWT.

[25] Mojarrad L.S., Rafe A. (2018). Effect of high-amylose corn starch addition on canning of yellow alkaline noodle composed of wheat flour and microbial transglutaminase: Optimization by RSM. Food Sci. Nutr..

[26] Li S., Tang D., Liu S., Qin S., Chen Y. (2020). Improvement of noodle quality: The effect of ultrasonic on noodles resting. J. Cereal Sci..

[27] Xing J.-j., Cheng L.-l., Feng S., Guo X.-n., Zhu K.-x. (2023). Humidity-controlled heat treatment of fresh spinach noodles for color preservation and storage quality improvement. Food Chem. X.

[28] Liu H., Guo X.N., Zhu K.X. (2022). Effects of freeze-thaw cycles on the quality of frozen raw noodles. Food Chem..

[29] Gwak N., Zogona D., Herrera Balandrano D.D., Beta T. (2026). Physicochemical Properties and Antioxidant Activity of Wild Rice–Wheat Noodles Enriched With Purple Sweet Potato Powder. J. Food Biochem..

[30] Tuersuntuoheti T., Wang Z., Duan M., Asimi S., Ren X., Wang Z., Zheng Y., Wu Y., Liang S., Zhang M. (2020). Noodle processing, storage time and cooking affect the antioxidant activities and phenolic compounds content of Qingke barley noodles. Int. J. Food Sci. Technol..

[31] Adeyemo I.M., Laleye P.O., Falade K.O. (2026). Enhancing pasta quality through substitution of wheat with acha, sorghum and *Cirina forda* powder. J. Food Meas. Charact..

[32] Yu W., Xu D., Li D., Guo L., Su X., Zhang Y., Wu F., Xu X. (2019). Effect of pigskin-originated gelatin on properties of wheat flour dough and bread. Food Hydrocoll..

[33] Qin W., Pi J., Zhang G. (2022). The interaction between tea polyphenols and wheat gluten in dough formation and bread making. Food Funct..

[34] Li Y., Xie L., Jiang X., Cai G., Zhu G., Zheng Z., Liu F. (2023). Effect of anthocyanins on mechanical and physicochemical properties of wheat dough. J. Cereal Sci..

[35] Cao Z., Liu Y., Zhu H., Li Y., Xiao Q., Yi C. (2021). Effect of soy protein isolate on textural properties, cooking properties and flavor of whole-grain flat rice noodles. Foods.

[36] Shang Y., Jiang T., Liu Y., Xu Y., Gao Y., Xiao H., Ma Y., Yang S., Wei Z. (2025). Effect of phytochemicals from grape seed on the improvement of noodle quality and the structure of wheat gluten. LWT.

[37] Krekora M., Nawrocka A. (2022). Effect of a polyphenol molecular size on the gluten proteins–polyphenols interactions studied with FT-Raman spectroscopy. Food Biophys..

[38] Jannasch A., Wang Y.-J., Lee S.-O., Liyanage R., McClung A.M. (2024). Polyphenol-mediated covalent bonds on glutelin structural changes in rice with different bran colors. J. Cereal Sci..

[39] Balestra F., Cocci E., Pinnavaia G., Romani S. (2011). Evaluation of antioxidant, rheological and sensorial properties of wheat flour dough and bread containing ginger powder. LWT.

[40] Li L., Zhou W., Wu A., Qian X., Xie L., Zhou X., Zhang L. (2022). Effect of ginkgo biloba powder on the physicochemical properties and quality characteristics of wheat dough and fresh wet noodles. Foods.

[41] Xu J., Li X., Chen J., Dai T., Liu C., Li T. (2021). Effect of polymeric proanthocyanidin on the physicochemical and in vitro digestive properties of different starches. LWT.

[42] Chumsri P., Panpipat W., Cheong L.Z., Nisoa M., Chaijan M. (2022). Comparative evaluation of hydrothermally produced rice starch–phenolic complexes: Contributions of phenolic type, plasma-activated water, and ultrasonication. Foods.

[43] Zhang Z., Tian J., Fang H., Zhang H., Kong X., Wu D., Zheng J., Liu D., Ye X., Chen S. (2020). Physicochemical and digestion properties of potato starch were modified by complexing with grape seed proanthocyanidins. Molecules.

[44] Echave J., Seyyedi-Mansour S., Donn P., Jorge A.O.S., Cassani L., Barros L., Prieto M.A. (2024). Starch–Polyphenol Interactions: Impact on Food Structure and Starch Digestibility. Proceedings.

[45] Zeng X., Zheng B., Xiao G., Chen L. (2022). Synergistic effect of extrusion and polyphenol molecular interaction on the short/long-term retrogradation properties of chestnut starch. Carbohydr. Polym..

[46] Han X., Zhang M., Zhang R., Huang L., Jia X., Huang F., Liu L. (2020). Physicochemical interactions between rice starch and different polyphenols and structural characterization of their complexes. LWT.

[47] Tang P., Zhang S., Meng L., Wang Z., Yang Y., Shen X., Tang X. (2023). Effects of different content of EGCG or caffeic acid addition on the structure, cooking, antioxidant characteristics and in vitro starch digestibility of extruded buckwheat noodles. Int. J. Biol. Macromol..

[48] Wang J., Feng L., Shen S., Zhang J., Wang Z., Zhang Y., Wang T., Wang H. (2024). Effect of Grape Seed Procyanidins on Rheological Properties of Wheat Dough and Noodles Quality. Shipin Gongye Ke-Ji.

[49] Xu M., Du J., Hou G.G., Du X. (2023). Effect of tea extract on starch gelatinisation, gluten aggregation and quality characteristics of dry yellow alkaline noodle. Int. J. Food Sci. Technol..

[50] Chen T., Xu R., Gao X., Wei S., Cheng Q., Meng L., Zhang J., Cheng Y. (2025). Effect of black tea extract on fresh rice noodles: Multiple quality attributes and underlying mechanism. Food Chem. X.

[51] Tuersuntuoheti T., Wang Z., Wang Z., Duan M., Zheng Y., Wu Y., Liang S., Li X., Zhang M. (2019). Microbes, bioactive compounds, quality characteristics, and structural changes during the storage of Qingke barley fresh noodles. J. Food Process. Preserv..

[52] Tiga B.H., Kumcuoglu S., Vatansever M., Tavman S. (2021). Thermal and pasting properties of Quinoa—Wheat flour blends and their effects on production of extruded instant noodles. J. Cereal Sci..

[53] Chen S.-X., Ni Z.-J., Thakur K., Wang S., Zhang J.-G., Shang Y.-F., Wei Z.-J. (2021). Effect of grape seed power on the structural and physicochemical properties of wheat gluten in noodle preparation system. Food Chem..

[54] Lee D.S., Kim Y., Song Y., Lee J.H., Lee S., Yoo S.H. (2016). Development of a gluten-free rice noodle by utilizing protein-polyphenol interaction between soy protein isolate and extract of *Acanthopanax sessiliflorus*. J. Sci. Food Agric..

[55] Kaur S., Sharma S., Singh B., Dar B.N. (2015). Effect of extrusion variables (temperature, moisture) on the antinutrient components of cereal brans. J. Food Sci. Technol..

[56] Atamyradova N., Özkılıç S.Y., Arslan D. (2024). Blanching of olive fruits before storage at different conditions: Effects on oil yield, lipase activity and oxidation. J. Agric. Food Res..

[57] Özkılıç S.Y., Arslan D. (2022). Acidic and enzymatic pre-treatment effects on cold-pressed pumpkin, terebinth and flaxseed oils. Grasas y Aceites.

[58] Alasalvar C., Shahidi F. (2009). Natural antioxidants in tree nuts. Eur. J. Lipid Sci. Technol..

[59] Stagos D. (2019). Antioxidant activity of polyphenolic plant extracts. Antioxidants.

[60] Perron N.R., Brumaghim J.L. (2009). A review of the antioxidant mechanisms of polyphenol compounds related to iron binding. Cell Biochem. Biophys..

[61] Karakaya S.N., Özkılıç S.Y., Arslan D. (2026). Effect of Combined Pretreatments on Yield and Quality of Cold-Pressed Pomegranate Seed Oil. Foods.

[62] Li T., Wang H., Zhang H., Cheng C., Wang Z., Zhou S., Wang K., Yang S. (2025). The characterization of sensory properties, aroma profile and antioxidant capacity of noodles incorporated with asparagus tea ultra-micro powder. Food Chem. X.

